# Supplementary material for: GP88/PGRN Serum Levels Are Associated with Prognosis for Oral Squamous Cell Carcinoma Patients
Source: Biology (Basel). 2021 May 4;10(5):400. doi: 10.3390/biology10050400 (PMC8147813; doi:10.3390/biology10050400)
Supplement: Supplementary file 1 [file biology-10-00400-s001.zip › biology-1176219-supplementary.pdf]

biology

MDPI

Supplementary Materials

GP88/PGRN Serum Levels Are Associated with Prognosis for Oral Squamous Cell Carcinoma Patients

Thomas Greither, Tina Steiner, Matthias Bache, Ginette Serrero, Sven Otto, Helge Taubert, Alexander Eckert and Matthias Kappler

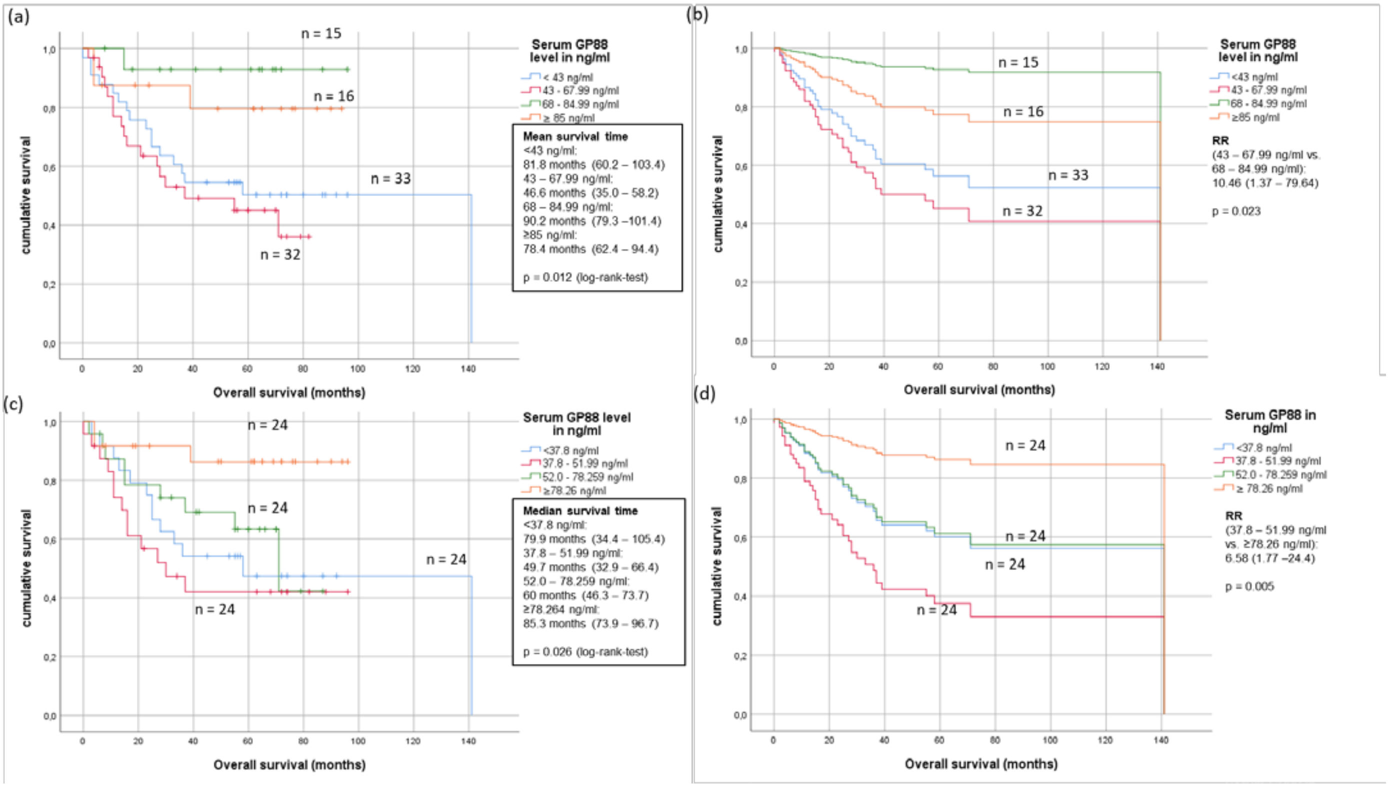

Figure S1. Kaplan-Meier (**a**;**c**) and multivariate Cox Regression (**c**;**d**) survival analyses of serum GP88 levels in OSCC patients with subgroups according to the Youden index optimized subgroups (**a**) and (**b**) and quartiles (**c**) and (**d**). Multivariate Cox Regression analyses were adjusted according to T, N and G.

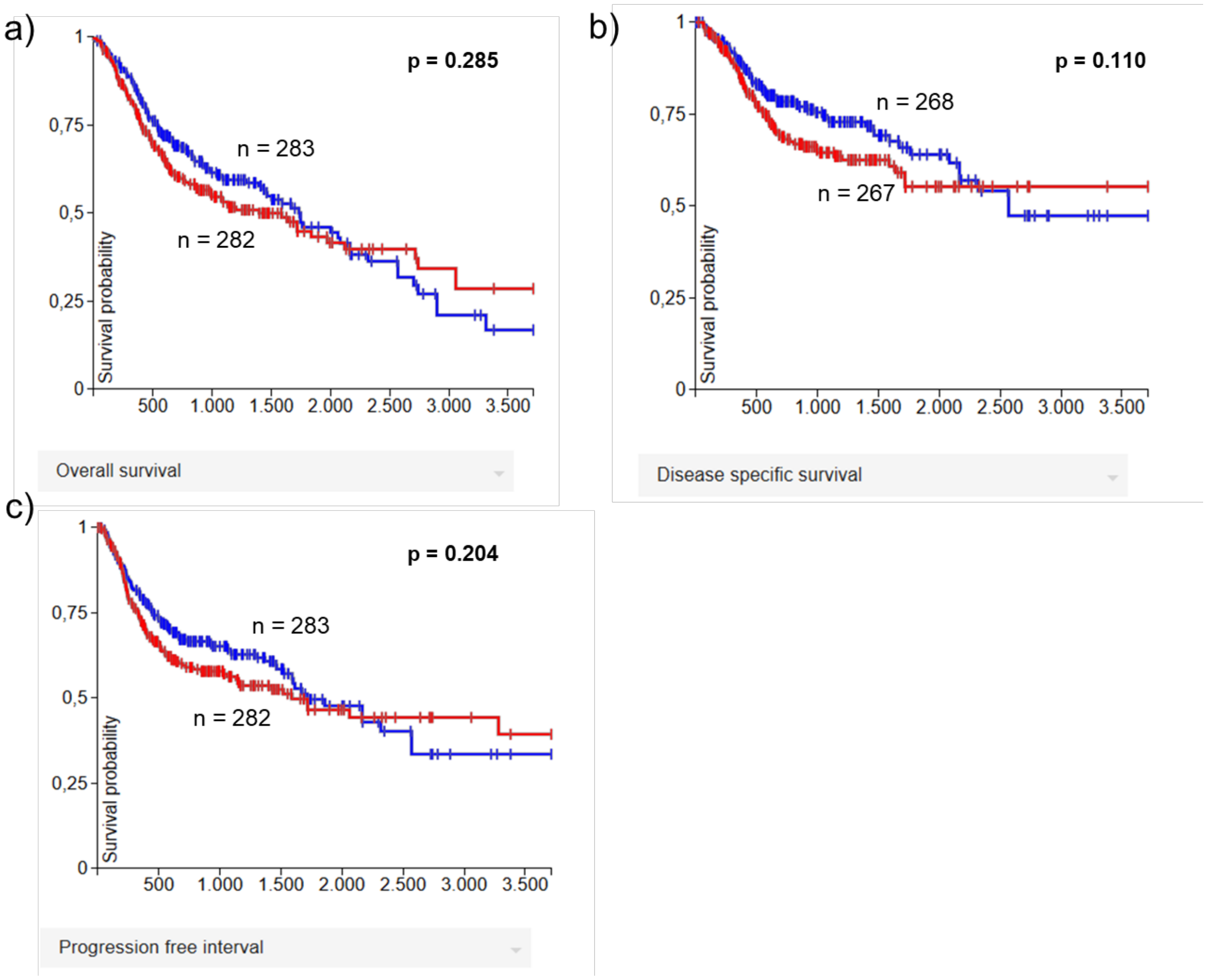

Figure S2. Kaplan-Meier survival analyses of the PGRN transcript in head and neck squamous cell carcinoma including OSCC patients regarding the (**a**) overall survival, (**b**) the disease-specific survival or (**c**) the progression free interval using TCGA data. Kaplan-Meier curves were drawn with the help of the USCS Xena program (University of California Santa Cruz; https://xenabrowser.net).

Table S1. Multivariate Cox Regression analyses regarding the impact of T stage, N status and Grading on patients survival in addition to serum GP88 levels (cut-off value: 68 ng/ml). RR = relative risk.

Parameter	n	RR	95% CI	p
T1	21		reference	
T2	34	1.29	0.43 - 3.83	0.65
T3	20	2.08	0.67 - 6.46	0.20
T4	21	2.02	0.61 - 6.63	0.25
N0	60		reference	
N1	12	2.83	0.53 - 6.30	0.34
N2+	24	1.80	0.80 - 3.98	0.16
G1	14		reference	
G2	59	2.13	0.80 - 5.68	0.13
G3	23	1.35	0.49 - 3.71	0.56

Table S2. Uni- and multivariate Cox Regression analyses on the impact of serum GP88 levels in male and female OSCC patients.

		univariate Cox Regression analysis			multivariate Cox Regression analysis		
		p	RR [95%CI]	*n*	*p*	RR [95% CI]	*n*
male	GP88 serum level <68.0 ng/ml	0.013	4.55 [1.37–15.13]	46	0.028	3.98 [1.16–13.61]	46
	GP88 serum level ≥68.0 ng/ml		reference	23		reference	23
female	GP88 serum level <64.3 ng/ml	0.113	5.32 [0.67–42.08]	18	0.154	4.97 [0.55–45.10]	18
	GP88 serum level ≥64.3 ng/ml		reference	9		reference	9

Table S3. Uni- and multivariate Cox Regression analyses on the impact of serum GP88 levels in patients with T1/2 or T3/4 tumors.

		univariate Cox Regression analysis			multivariate Cox Regression analysis		
		p	RR [95%CI]	n	p	RR [95% CI]	n
T1/2	GP88 serum level <44.1 ng/ml	0.017	3.72 [1.27–10.90]	21	0.001	8.22 [2.32–29.10]	21
	GP88 serum level ≥44.1 ng/ml		reference	34		reference	34
T3/4	GP88 serum level <63.4 ng/ml	0.037	8.42 [1.13–62.62]	29	0.058	7.17 [0.94–54.96]	29
	GP88 serum level ≥63.4 ng/ml		reference	12		reference	12

Table S4. Uni- and multivariate Cox Regression analyses on the impact of serum GP88 levels in patients with N0 or N+ tumors.

		univariate Cox Regression analysis			multivariate Cox Regression analysis		
		p	RR [95%CI]	n	p	RR [95% CI]	n
N0	GP88 serum level <63.0 ng/ml	0.016	4.58 [1.33–15.61]	35	0.037	3.98 [1.08–14.58]	35
	GP88 serum level ≥63.0 ng/ml		reference	25		reference	25
N+	GP88 serum level <68.0 ng/ml	0.097	5.54 [0.73–41.85]	28	0.116	5.69 [0.65–49.82]	28
	GP88 serum level ≥68.0 ng/ml		reference	8		reference	8

Table S5. Uni- and multivariate Cox Regression analyses on the impact of serum GP88 levels in patients with or without subsequent radiotherapy.

		univariate Cox Regression analysis			multivariate Cox Regression analysis		
		p	RR [95%CI]	n	p	RR [95% CI]	n
no RT	GP88 serum level <63.0 ng/ml	0.006	5.54 [1.63–18.87]	40	0.006	5.81 [1.67–20.24]	40
	GP88 serum level ≥63.0 ng/ml		reference	30		reference	30
RT	GP88 serum level <42.0 ng/ml		reference	12		reference	12
	GP88 serum level ≥42.0 ng/ml	0.095	2.4 [0.86–6.68]	14	0.109	2.98 [0.79–11.31]	14
